# RETRACTION: Comparison of Therapeutic Effects Between Artificial Dermis Combined With Autologous Split‐Thickness Skin Grafting and Autologous Intermediate‐Thickness Skin Grafting Alone in Severely Burned Patients: A Prospective Randomised Study

**DOI:** 10.1111/iwj.70652

**Published:** 2025-04-23

## Abstract

**RETRACTION:** ShangF. , LuY.H. , GaoJ. , and HouQ. , “Comparison of Therapeutic Effects Between Artificial Dermis Combined With Autologous Split‐Thickness Skin Grafting and Autologous Intermediate‐Thickness Skin Grafting Alone in Severely Burned Patients: A Prospective Randomised Study,” International Wound Journal 18, no. 1 (2021): 24–31, 10.1111/iwj.13518.33124156 PMC7948658

The above article, published online on 30 October 2020, in Wiley Online Library (http://onlinelibrary.wiley.com/), has been retracted by agreement between the journal Editor in Chief, Professor Keith Harding; and John Wiley & Sons Ltd. Following an investigation by the publisher, all parties have concluded that this article was accepted solely on the basis of a compromised peer review process. The editors have therefore decided to retract the article. The authors did not respond to our notice regarding the retraction.

**RETRACTION:** F. Shang , Y.H. Lu , J. Gao , and Q. Hou , “Comparison of Therapeutic Effects Between Artificial Dermis Combined With Autologous Split‐Thickness Skin Grafting and Autologous Intermediate‐Thickness Skin Grafting Alone in Severely Burned Patients: A Prospective Randomised Study,” International Wound Journal 18, no. 1 (2021): 24–31, 10.1111/iwj.13518.33124156 PMC7948658

The above article, published online on 30 October 2020, in Wiley Online Library (http://onlinelibrary.wiley.com/), has been retracted by agreement between the journal Editor in Chief, Professor Keith Harding; and John Wiley & Sons Ltd. Following an investigation by the publisher, all parties have concluded that this article was accepted solely on the basis of a compromised peer review process. The editors have therefore decided to retract the article. The authors did not respond to our notice regarding the retraction.

